# Approaches to augment vascularisation and regeneration of the adult heart via the reactivated epicardium

**DOI:** 10.21542/gcsp.2016.28

**Published:** 2016-12-30

**Authors:** Owen J. Duffey, Nicola Smart

**Affiliations:** Department of Physiology, Anatomy and Genetics, University of Oxford, Oxford, UK

## Abstract

Survival rates following myocardial infarction have increased in recent years but current treatments for post-infarction recovery are inadequate and cannot induce regeneration of damaged hearts. Regenerative medicine could provide disease-reversing treatments by harnessing modern concepts in cell and developmental biology. A recently-established paradigm in regenerative medicine is that regeneration of a tissue can be achieved by reactivation of the coordinated developmental processes that originally formed the tissue. In the heart, the epicardium has emerged as an important regulator of cardiac development and reactivation of epicardial developmental processes may provide a means to enable cardiac regeneration. Indeed, in adult mouse hearts, treatment with thymosin β4 and other drug-like molecules reactivates the epicardium and improves outcomes after myocardial infarction by inducing regenerative paracrine signalling, neovascularisation and *de novo* cardiomyocyte production. However, there are considerable limitations to current methods of epicardial reactivation that prevent direct translation into clinical practice. Here, we describe the rationale for targeting the epicardium and the successes and limitations of this approach. We consider how several recent advances in epicardial biology could be used to overcome these limitations. These advances include insight into epicardial signalling and heterogeneity, epicardial modulation of inflammation and epicardial remodelling of extracellular matrix.

## Introduction

Coronary heart disease is a leading cause of worldwide morbidity and mortality^1^. Despite improved survival rates following myocardial infarction (MI), current treatments are unable to reverse loss of cardiac function after MI^1–3^. By harnessing modern concepts in cell and developmental biology, regenerative medicine could provide novel treatments to repair diseased hearts^4^.

The adult mammalian heart has long been regarded as non-proliferative and terminally differentiated but recent evidence has demonstrated that cardiomyocytes are capable of low level turnover, particularly after MI^5–7^. However, the limited proliferative capacity of the mammalian heart, beyond early neonatal stages^8^, is grossly insufficient to regenerate the ∼1 billion cardiomyocytes lost in a typical MI^9^. Moreover, regeneration of myocardial tissue is a complex process requiring appropriate integration of newly produced cardiomyocytes, production and integration of non-cardiomyocyte cell types, regenerative paracrine signalling, and moderation of inflammation that promotes excessive fibrosis. Therefore, even if cardiomyocytes could be easily replenished, such approaches, in isolation, are unlikely to induce sufficient regeneration.

Several different approaches to cardiac regeneration have been studied (Table 1), both in preclinical and clinical trials, described in^10^. These are principally based upon inducing cardiomyocyte cell cycle re-entry^11^, directly reprogramming cardiac-resident non-myocytes (fibroblasts)^12^ or cell therapy-based strategies. The latter approach has applied a range of somatic^13^ and haematopoietic^14^ cell types, resident cardiac progenitor cells^15–18^, embryonic (ESCs) or induced pluripotent stem cells (iPSCs). Despite numerous multi-centre clinical trials of transplanted autologous bone marrow stem cells, transdifferentiation into cardiac muscle appears not to occur and any clinical improvement is modest^19^, attributable to the local release of paracrine factors that enhance repair, attenuate fibrosis and improve cardiac functional recovery. Resident cardiac progenitor cells were anticipated by many to confer superior benefit.

**Table 1:** An overview of different approaches to cardiac regeneration, including epicardial reactivation. Abbreviations: AAV9, adeno-associated virus 9; BMP4, bone morphogenetic protein 4; CDC, cardiosphere-derived cell; CM, cardiomyocyte; CPC, cardiac progenitor cell; ESC-CM, embryonic stem cell-derived cardiomyocyte; FSTL1, Follistatin-like 1; HGF, hepatocyte growth factor; iCM, induced cardiomyocyte-like cells; IGF-1, insulin-like growth factor 1; iPSC-CM, induced pluripotent stem cell-derived cardiomyocyte; LVEF, left ventricular ejection fraction; MI, myocardial infarction; miR, microRNA; modRNA, modified RNA; NRG-1, neuregulin-1; Tβ4, thymosin β4.

Approach	Advantages	Disadvantages	Notable examples
**Induce cardiomyocyte cell cycle re-entry**			
Induce proliferation of mature CMs to replace CMs lost in heart disease.	• Autologous (eliminates risk of rejection and requirement for immunosuppression). • No requirement for stimulating CM differentiation and maturation. • New CMs are likely to have good mechanical, vascular and electrical integration.	• Potential off-target effects of treatment (particularly oncogenesis). • Difficult to achieve the magnitude of required CM proliferation for clinically meaningful benefit. • Inherent difficulties of gene therapy approaches – non-genetic approaches (eg. paracrine factors) more desirable.	• Progressive improvement in infarct size and cardiac functional parameters occurred in cyclin D2 transgenic mice^29^. • Activation of the Hippo/YAP promitogenic signalling pathway improved heart function and survival after MI^11^. • AAV9 gene transfer of miR-590-3p and miR-199a-3p stimulated CM proliferation, reduced infarct size and improved cardiac functional parameters after MI^30^. • Inhibition of miR-15 induced cardiac proliferation and modestly improved cardiac function^31^. • In a small clinical trial, infusion of human recombinant NRG-1 was well-tolerated and improved cardiac function. NRG-1 has pro-proliferative effects *via* the ErbB2/ErbB4 receptor – oncogenic potential is an important concern^32^. • Deletion of Meis1, a cardiomyocyte cell cycle regulator, extends the postnatal window of proliferation and regeneration, although the ability to induce the effect in the adult heart was not reported^33^. • Reconstituting the potent cardiogenic activity of FSTL1 in an epicardial patch promoted myocardial regeneration following MI, in mouse and pig^34^.
**Cell therapy**			
Produce ESC- or iPSC-derived CMs *in vitro* and deliver to the myocardium.	• Successful long-term engraftment of substantial numbers of ESC-CMs has been achieved in animal models. • Potentially highly reproducible. • Highly specific effects.	• ESC-CMs: allogeneic and ethical concerns. • iPSC-CMs: logistical and regulatory concerns. • Possibility of teratoma formation. • Stem cell-derived CMs are immature - improper mechanical, electrical and vascular integration.	• Human ESC-CMs could be produced on a clinical scale. Delivery of human ESC-CMs to infarcted non-human primate hearts produced extensive remuscularisation^25^.
Isolate resident or non-resident cardiac progenitor cells, expand *in vitro*and deliver to the myocardium.	• Autologous CPCs can be obtained from biopsies collected during surgery. • Clinical trials have demonstrated safety (but safety might be compromised at the higher engraftment rates that would be desirable for improved treatment).	• Limited efficacy and inconsistent results in clinical trials. • Unclear mechanism of action – paracrine effects are likely to be important, in which case paracrine factor therapy may have advantages over cell-based therapy. • CMs may not appropriately integrate.	• In a randomised, open-label phase 1 trial (‘SCIPIO’), c-kit^+^ CPC-treated patients had a small improvement in LVEF^20^. However, *The Lancet* has issued an “expression of concern” regarding data integrity.^21^. In independent murine studies, c-kit^+^ CPCs made only minimal^23^, or no^24^, contribution to CMs. • In the ‘CADUCEUS’ trial, intracoronary infusion of autologous CDCs after MI was safe and reduced scar mass and regional contractile dysfunction; however the patient cohort was small^22^.
**Enhance activity of endogenous CPCs**			
Stimulate resident CPCs *in situ* to increase regenerative activity.	• Potential to regenerate a range of cell types in addition to CMs. • Cell-free (less costly, fewer logistical difficulties and perhaps safer than cell-based therapies).	• Potential off-target effects. • Robust regenerative technique not yet described.	• “Priming” with Tβ4 activated the epicardium, resulting in *de novo* CMs and improved functional cardiac parameters^28^. • Intra-myocardial injection of modRNA encoding VEGF-A enhanced mobilisation of epicardial progenitor cells and improved heart function and survival in mice^35^. •*In vivo* injection of HGF and IGF-1 into murine hearts to mobilise and amplify resident c-kit^+^ CPCs improved function and survival after MI^36^.
**Direct reprogramming of non-cardiomyocytes**			
Use cardiac reprogramming factors to induce conversion of non-cardiomyocytes (e.g. fibroblasts) into iCMs *in vivo*.	• Convert excessive fibroblasts (which induce scarring) into functional iCMs. • Cell-free (less costly, fewer logistical difficulties and perhaps safer than cell-based therapies).	• Requires gene transfer by integrating viruses. • Low reprogramming efficiency. • Functional integration of iCMs is not established. • Difficult to confirm fibroblast-to-iCM reprogramming *in vivo* in patients. • Requires development of appropriate models for translation into humans.	• Retroviral gene transfer and expression of *Gata4*, *Mef2c*, and *Tbx15* (GMT)^12^ or additionally *Hand2* (GHMT)^37^ induced reprogramming of fibroblasts into CMs and improved cardiac function after MI in mice. • Reprogramming with a combination of *Hand2*, *Nkx2.5*, *Gata4*, *Mef2c*, and *Tbx5* (HNGMT) has a >50-fold higher efficiency than with GMT alone^38^. • Enhanced efficiency of reprogramming with Tβ4 treatment^39^, illustrating potential benefit of combining approaches.

Whereas the findings from the SCIPIO trial appear promising^20^, concerns have been raised over some of the data^21^. Early evidence from the CADUCEUS trial^22^ suggests that cardiosphere-derived autologous stem cells confer modest functional improvement. In contrast, recent studies in rodents, where lineage tracing technologies can be applied, revealed minimal contribution of c-kit+ cells to cardiac regeneration^23,24^; indeed, the value of c-kit as a marker of cardiac stem cell potential was questioned when c-kit positive cells were revealed to be an endothelial cell population with no cardiomyogenic contribution^24^. A recent proof-of-concept study in a non-human primate model highlighted the safety and potential for extensive remuscularisation using human ESC-derived cardiomyocytes^25^. Given the ethical concerns that limit application of ESCs, the hopes of cardiac cell therapy may, therefore, depend upon improving functional maturation of iPSC-derived cardiomyocytes^26^.

An emerging paradigm in regenerative medicine is that repair of tissue can be achieved by reactivation of the coordinated developmental processes that originally formed the tissue^27^. Of note, the epicardium has been described as an important regulator of myocardial development and experimental treatments that reactivate embryonic epicardial processes improve outcomes after an experimental model of MI in adult mice^28^. However, these treatments have major limitations and are unlikely to be clinically useful in their current form. We discuss the rationale for targeting the epicardium and explore the successes and limitations of pre-clinical studies that demonstrate proof-of-principle of epicardial reactivation for cardiac regeneration. Moreover, we consider how recent evidence highlighting several novel concepts related to epicardial biology could help to overcome previous limitations. These avenues for future research may aid progress towards the ultimate aim of clinical induction of cardiac regeneration in the adult human.

## Why target the epicardium?

### The epicardium is essential for mammalian cardiac development

**Figure 1.**
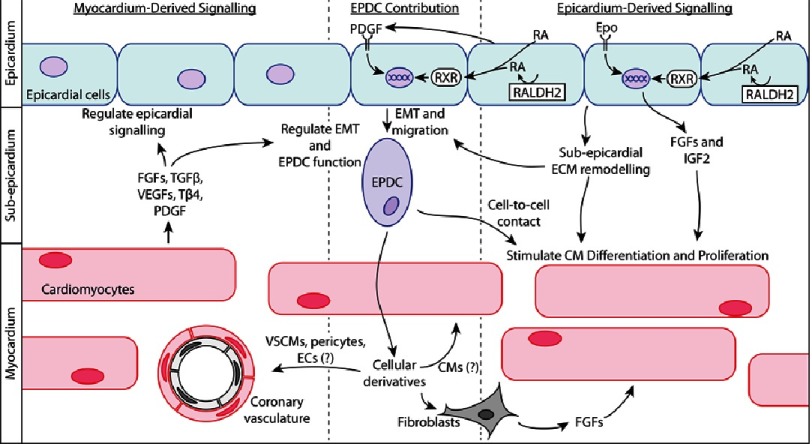
Epicardial cellular contribution and reciprocal epicardial-myocardial signalling are critical for cardiac development and may similarly determine epicardial potential for cardiac regeneration. Abbreviations: CM, cardiomyocyte; EC, endothelial cell; EMT, epithelial to mesenchymal transition; EPDC, epicardium-derived cell; Epo, erythropoietin; FGF, fibroblast growth factor; IGF2, insulin-like growth factor 2; PDGF, platelet-derived growth factor; RA, retinoic acid; RALDH2, retinaldehyde dehydrogenase 2; RXR, retinoid X receptor; Tβ4, thymosin β4; TGFβ, transforming growth factor β; VEGF, vascular endothelial growth factor; VSMCs, vascular smooth muscle cells.

The epicardium develops principally from the mesodermal proepicardial organ soon after cardiac looping^40,41^. The epicardium then makes essential cellular and signalling contributions to cardiac development (Figure 1).

The cellular contribution is mediated by a subpopulation of epicardial cells that undergoes epithelial-to-mesenchymal transition (EMT) to produce epicardium-derived cells (EPDCs). EPDCs then migrate into the myocardium and differentiate into several cardiac lineages^41^. In development, it is widely accepted that EPDCs make essential contributions to cardiac interstitial fibroblasts, along with adventitial fibroblasts, vascular smooth muscle cells and pericytes of the coronary vessels^41^. However, the contribution of EPDCs to cardiomyocyte and coronary endothelial lineages is more controversial. Modern fate map studies primarily use *Cre*-*LoxP*-based genetic lineage tracing, whereby Cre recombinase is expressed with a lineage-specific gene to induce genetic recombination and consequent lineage-specific expression of a reporter gene (eg. enhanced green fluorescent protein or β-galactosidase).

These genetic changes are inherited by daughter cells (‘indelible labelling’) and can be assessed by histological techniques. Using *Tbox18* (*Tbx18*) and *Wilms’ tumour-1* (*Wt1*) as epicardial marker genes for such genetic lineage tracing, a subset of cardiomyocytes was found to be derived from Tbx18^+^ or Wt1^+^ cells^42,43^. However, an important consideration in genetic lineage tracing is the specificity and sensitivity of the marker gene. In this regard, *Tbx18* is expressed in epicardial cells (high sensitivity) but is also expressed in the interventricular septum and left ventricular cardiomyocytes (low specificity)^44^. Similarly, a tamoxifen-inducible *Wt1*CreERT2 drives incomplete epicardial recombination (low sensitivity) and *Wt1* is reported to be expressed in coronary endothelial cells (low specificity)^45,46^. Since *Tbx18* and *Wt1* expression is not strictly limited to the epicardium, cells labelled by such genetic lineage tracing could derive from sources other than epicardial cells, and therefore the epicardial contribution to the cardiomyocyte lineage has been disputed^44,45^.

Analogous inadequacy of marker genes may underlie the controversy surrounding the (pro)epicardial contribution to the coronary endothelium^47^. A substantial contribution to the coronary endothelium is made by recently-identified proepicardial sub-populations that express neither *Wt1* nor *Tbx18*^47^, thus, previous studies using these marker genes did not include the relevant sub-population (low sensitivity) and therefore the previous conclusion of a lack of (pro)epicardial contribution to the coronary endothelium may be incorrect^42–44,47–49^. While alternative sources, notably the sinus venosus^48^ and endocardium^50^, have also been described, those endothelial cells contributed from the proepicardium appear to derive from a distinct subpopulation, characterised by expression of *Scleraxis* (*Scx*) and *Semaphorin3D* (*Sema3D*)^47^.

In addition to cellular contributions, the epicardium also participates in reciprocal, bi-directional signalling between the epicardium and myocardium^51^ (Figure 1). Epicardium-derived signalling functions include promoting cardiomyocyte proliferation and differentiation and stimulating coronary vascularisation^27^; as such, epicardium-derived factors are essential for the formation of mature myocardium^52^. Retinoic acid and erythropoietin signalling within the epicardium stimulates epicardial production of fibroblast growth factor (FGF) family members and insulin-like growth factor 2 (IGF2), which govern normal development in the underlying myocardium^53–60^. Epicardial signalling also stimulates epicardial EMT, facilitating epicardial cellular contribution to the myocardium^61,62^. Epicardial EMT facilitates direct contact between EPDCs and myocardial cells, which enhances cardiomyocyte proliferation, maturation and alignment^63,64^. Finally, modification of extracellular matrix (ECM) composition by the epicardium and EPDCs has important effects on both epicardial and myocardial development^51,65–67^.

Conversely, the myocardium also signals to the epicardium to control epicardial activities, such as EMT and migration and differentiation of EPDCs^27,68^. This occurs by myocardial secretion of signalling factors, which include FGF family members^69^, transforming growth factor β (TGFβ)^70,71^, vascular endothelial growth factors (VEGFs)^72^, thymosin β4 (Tβ4)^73^ and platelet-derived growth factor (PDGF)^61,74^. Thus, the epicardium and myocardium form a reciprocal signalling unit that is essential for cardiac development.

### The epicardium is essential for non-mammalian cardiac regeneration

The epicardium is not only important in mammalian cardiac development but is also critical for cardiac regeneration in species such as zebrafish, which are able to fully recover after substantial cardiac injury. During zebrafish cardiac regeneration, the epicardium re-expresses embryonic genes *Retinaldehyde dehydrogenase 2* (*Raldh2*), a regulator of retinoic acid synthesis, and *Tbx18* resulting in epicardial EMT and EPDC migration to vascularise the myocardium^75^. By contrast, in adult mammals, epicardial proliferation and signalling occurs after injury but, without therapeutic intervention, this is unable to invoke substantial regeneration^76,77^.

### Rationale for targeting the epicardium

Epicardial programmes underpin cardiac development and re-activation of these programmes mediates regeneration in zebrafish. However, mammalian epicardial reactivation requires enhancement to be therapeutically useful. Importantly, the epicardium can uniquely contribute to coordinated repair by providing a range of cell types and signalling factors.

## Prior successes and limitations in reactivating the quiescent adult mammalian epicardium

### Thymosin β4 treatment improves outcomes after myocardial infarction in mice

Thymosin β4 (Tβ4) is a 43-amino acid actin monomer-binding peptide, which is important for both systemic and coronary vascular development^73,78^. In the adult mammalian heart, Tβ4 treatment stimulates reactivation of the epicardium after MI and results in regenerative neovascularisation^79^, recapitulating its embryonic role. Furthermore, ‘priming’ by treatment with Tβ4 *before* MI enables EPDCs to form *de novo* cardiomyocytes^28^, and induces a robust neovascularisation^79,80^. In the short time frame of 28 days after MI, Tβ4 treatment reduced infarct volume and improved ejection fraction^28^. New cardiomyocytes must integrate properly into the myocardium in order to maintain structural integrity and avoid arrhythmogenic electrophysiological heterogeneity. After Tβ4 stimulation, epicardium-derived cardiomyocytes formed adherens and gap junctions, suggesting structural integration^28^; moreover, functional integration of the *de novo* cardiomyocytes was demonstrated by synchronous [Ca^2+^]_*i*_ transients between *de novo* and pre-exisiting cardiomyocytes^28^. However, given the complexities of coupling between cardiomyocytes, as well as other functional links such as proposed cardiomyocyte-fibroblast coupling^81^, more comprehensive assessment of *de novo* cardiomyocyte integration might be required in order to conclude complete integration.

### Limitations of Thymosin β4 and other treatments

EPDCs from the adult mouse after Tβ4 treatment are not molecularly identical to embryonic EPDCs, despite functional similarities^82^. Reactivated adult EPDCs display a heterogeneous molecular profile, defined by both cardiac progenitor and mesenchymal stem cell markers, including Sca-1, CD29, CD90, PDGFRβ and CD44, thus are significantly different from their embryonic counterparts obtained at mouse embryonic day 12.5 (E12.5), despite the common expression of the early embryonic epicardial gene *Wt1*^82^. Consequences of the molecular differences are unclear but this finding suggests that Tβ4 priming does not achieve true recapitulation of embryonic processes, which perhaps limits maximal therapeutic benefit.

In contrast to Tβ4 treatment *before* MI, Tβ4 treatment *after* MI does not produce *de novo* cardiomyocytes, although it does confer modest benefits, probably via paracrine effects^83^. This is a considerable limitation for translation into clinical practice because it presents a requirement for prophylactic treatment in order to regenerate *de novo* cardiomyocytes after MI. Although such prophylactic treatment is possible, particularly if at-risk patients were targeted, widespread long-term Tβ4 treatment may give cause for concern over issues such as safety, cost, patient compliance, and feasibility of frequent administration by necessarily non-oral routes.

Perhaps the most important limitation of Tβ4 treatment is low efficiency of *de novo* cardiomyocyte production from EPDCs (∼0.59% of Wt1^+^ EPDCs)^28^. In addition to Tβ4, two other drug-like molecules, prokineticin-2 and VEGF-A modified RNA (modRNA), are also able to reactivate the epicardium but they too have important limitations^35,84,85^. On balance, these experimental treatments demonstrate the principle of epicardial reactivation for cardiac regeneration but several difficulties prevent direct translation into clinical practice.

## Novel research avenues that could improve epicardial reactivation strategies

### Understanding the regulatory pathways that restrict epicardial cell behaviour

Extrapolating from our understanding of epicardial processes in development and from recent insights into their redeployment for regenerative benefit by zebrafish following injury, we can clearly appreciate the shortcomings of the cardiac healing response in mammals. Therapeutically instilling developmental or cardioregenerative mechanisms into the refractory mammalian heart may support a greater degree of muscle regeneration. As far as engaging epicardial involvement in regeneration is concerned, at least three key processes need to be targeted: (i) reactivation and restoration of pluripotency; (ii) EMT and inward migration; (iii) cell fate determination. While partial success in stimulating each of these steps has been achieved, notably with Tβ4, the extent, as previously stated, is inadequate, particularly of EMT and in directing cardiomyocyte cell fate. While screening for potent small molecules^86^ may lead to a breakthrough, candidate approaches, based on understanding embryonic and zebrafish mechanisms, may also prove fruitful. Taking EMT as an example, the principal drivers of epicardial EMT in the embryo are FGFs, notably basic FGF and TGFβ^68^, as discussed above. Furthermore, the regenerative capacity of zebrafish is FGF-dependent; expression of a dominant-negative FGF receptor blocks epicardial EMT, neovascularisation and regeneration^75^. Identifying suitable targets to achieve enhanced activation of these pathways in epicardial cells may be beneficial. Understanding the pathways that regulate cardiac cell differentiation during development may also reveal novel targets to enhance the complement of pro-regenerative cell types, at the expense of the predominant fibroblast fate.

### Understanding the intrinsic response of the epicardium to myocardial injury

Despite its recognised failure to regenerate, the adult mammalian heart displays an intrinsic response to injury which, to date, has only been superficially characterised. Whilst scarring, ventricular dilatation and hypertrophic responses are widely recognised^87^, other elements have been largely overlooked. One such endogenous response is the reactivation of the epicardium, in the form of re-expression of embryonic epicardial genes^28^ and expansion via proliferation^28^ and infiltration of haematopoietic cells^40^. These responses would logically appear to be beneficial, an attempt at self-repair, for example by secretion of pro-regenerative paracrine factors^77^ and induction of new vessel growth^79^. An appreciation of what these pathways contribute, and how they may be enhanced, may facilitate a greater degree of regeneration. However, not all intrinsic responses constructively influence repair, fibrosis being a prime example; while moderate fibrosis is essential, at least initially, to prevent cardiac rupture, excessive fibrosis leads to permanent scarring at the expense of myocardial replenishment^87^. Although some embryonic epicardial processes are redeployed^27^, others are either ineffectively induced or even actively suppressed. A notable, recently-identified example of this is expression of the secreted protein, Follistatin-like 1 (FSTL1)^34^. FSTL1 is a potent cardiogenic factor that is actively expressed by embryonic and adult epicardial cells. Curiously, epicardial FSTL1 declines following myocardial infarction but application of human FSTL1 via an epicardial patch was able to stimulate cardiomyocyte proliferation, to improve cardiac function and survival, in mouse and swine MI models^34^. These findings suggest that the loss of epicardial FSTL1 is a maladaptive response to injury, and that its restoration can reverse myocardial death and remodelling following MI. Thus, understanding endogenous responses empowers regenerative strategies but requires additional insight into whether to therapeutically enhance or override intrinsic mechanisms.

### The epicardium is a complex heterogeneous structure

The epicardium was, for a long time, regarded as a simple mesothelial layer with little heterogeneity, but recent work indicates hereto unappreciated complexities^41,47^. Epicardial sub-populations have been classified according to factors such as activation by signalling molecules (e.g. Notch or Tβ4) or expression of molecular markers (e.g. Wt1, Sca-1 or c-kit)^27,28,82,88^. However, one limitation of the literature is that different studies often assess different activating factors, molecular markers or developmental time points. The extent of overlap between different populations reported in separate studies is, therefore, not immediately apparent. For example, there is considerable molecular heterogeneity among the EPDCs that are reactivated by Tβ4^82^ and it is therefore likely that some of the Tβ4-stimulated epicardial cells could also be classified into sub-populations described elsewhere on the basis of other distinguishing factors. A detailed understanding of the different cell types that populate the epicardium might allow for more precise targeting of sub-populations relevant to regeneration. The first study to systematically characterise epicardial heterogeneity, using a single cell transcriptomic approach, confirmed that at least three distinct sub-populations of tcf21+ epicardial cells exist in zebrafish^89^. The specific functional roles of these sub-populations in cardiac development, homeostasis and regeneration remains to be explored and, crucially, the extent to which this heterogeneity is conserved, or potentially more complex, in mammals remains to be determined.

Assessment of murine epicardial structure has recently revealed additional heterogeneity in the form of clusters of CD45^+^ Wt1^−^ haematopoietic cells, encased in ECM, which resemble stem cell niches^40^. After MI, the CD45^+^ cells proliferated, encapsulating ECM was degraded by matrix metalloproteinases, and proliferative CD45^+^ cells were released into the underlying myocardium. Although the precise extent of this movement into the myocardium was not assessed, these results hint at a possible role for the CD45^+^ cells that deserves further investigation^40^, specifically to investigate whether the CD45^+^ cells contribute adversely or beneficially to cardiac regeneration. This study both challenges the prevailing dogma that the epicardium derives solely from the proepicardial organ and identifies a novel sub-population of epicardial cells that might be a useful therapeutic target.

### Controlling the inflammatory response to cardiac injury

In zebrafish and newts, regeneration occurs with only short-lived fibrosis. By contrast, MI in adult mammalian hearts results in inflammation, fibrosis and permanent scarring^90^. Regeneration of the adult mammalian heart may, therefore, require moderating the response away from excessive inflammation and fibrosis, which preclude regeneration, and towards cellular regeneration and integration.

Although use of Tβ4, prokineticin-2 and VEGF-A modRNA to reactivate epicardial developmental programmes is beneficial, it does not necessarily follow that epicardial activity, in the absence of developmental reactivation, is beneficial. Indeed, it was reported that epicardial activity may underlie, at least in part, the inflammatory fibrosis of the endogenous response to MI^91^. After identifying the CCAAT/enhancer binding protein (C/EBP) transcription factor family as important in epicardial activation, it was found that viral gene transfer of dominant-negative mutant C/EBP improved ejection fraction and fibrosis after MI and reduced local neutrophil count; however, the causal link between lowering the inflammatory infiltrate and improving outcome was not directly demonstrated^91^.

Thymosin β4 may also be useful for controlling inflammation. One study found that treatment of mice after MI with a biologically-occurring form of Tβ4 (Tβ4-sulfoxide) increased the local macrophage count at day 2 post-MI but reduced the macrophage count at day 7 compared to control^92^. In combination with the finding of reduced infarct volume after Tβ4 treatment, these results were interpreted to mean that Tβ4-sulfoxide hastens early phagocytic debris removal after MI and enhances subsequent clearance of immune cells. Although the epicardium may not have a direct role in these anti-inflammatory effects, these findings, along with its proven role in cardiomyocyte protection^93^, suggest that Tβ4 might be clinically useful for prevention of cardiac scarring, even if its role in stimulation of production of *de novo* cardiomyocytes by EPDCs is limited^80,92,94^.

The concept of beneficial effects of early macrophages in cardiac regeneration is supported by recent evidence showing that macrophage depletion prevents the regeneration that normally occurs in neonatal mice after MI^95^. The pro-regenerative macrophages had pro-angiogenic effects, although the underlying mechanisms remain unclear. Molecular profiling of the early macrophages revealed no clear bias towards M1-type or M2-type macrophages, instead pointing to a transient, pro-regenerative phenotype and secretion of distinct soluble factors that may facilitate myocardial regeneration. Although these findings identify a potentially important new macrophage subset, the non-canonical characteristics of the pro-regenerative neonatal macrophages might limit translation into clinical therapy^96^.

Thus, inflammation, fibrosis and scarring remain as major obstacles to cardiac regeneration, and the underlying mechanisms are complex. Improved understanding of the inflammatory response to cardiac injury, including the role played by the epicardium, might enable targeted modification of adverse fibrosis while preserving beneficial debris removal by macrophages^97,98^.

### Epicardium-controlled remodelling of the extracellular matrix

The ECM is a dynamic network of fibrous and non-fibrous proteins that can control cell function through several signalling mechanisms^65,99^. In development, the ECM has important effects on the epicardium and elsewhere in the heart^99^. For example, binding of epicardial α4-integrin to ECM ligands in the sub-epicardium inhibits EMT and migration, and influences epicardial cell differentiation^66^. Moreover, remodelling of the ECM recapitulates embryonic programmes to facilitate regeneration of limbs, tails and fins in fish and amphibians^100–103^.

Recently it was suggested that dynamic spatiotemporal ECM remodelling by the epicardium plays an important role in cardiac regeneration in zebrafish and newts^104,105^. *In situ* hybridisation and transgenic reporter analyses revealed that deposition of fibronectin-1 and fibronectin-1b by epicardial cells in zebrafish was dynamically upregulated during heart regeneration^104^. Furthermore, concomitant expression of integrin β3 and αV also occurred in cardiomyocytes, perhaps facilitating the fibronectin signalling to enhance mobilisation and integration of cardiomyocytes. Importantly, fibronectin-1-defective zebrafish displayed impaired myocardial regeneration and increased fibrosis^104^.

These findings suggest that the ECM acts as an important intermediate messenger for regenerative epicardium-to-myocardium signalling. Similarly, DNA microarray profiling and subsequent gene ontology analysis has identified an enrichment of genes associated with ECM in the regenerating hearts of newts and zebrafish^105^. Of particular interest were the ECM components hyaluronic acid, tenascin C and fibronectin, which were upregulated in the epicardium of the regenerating newt heart. Tenascin C induced cell cycle re-entry of primary cultured newt cardiomyocytes and may facilitate migration with ‘counter-adhesive domains’. It was speculated that the ‘regeneration-specific matrix’ may also facilitate migration by altering tissue stiffness through hyaluronic acid-dependent ECM hydration^105^.

However, while these studies show that ECM remodelling is important in newt and zebrafish cardiac regeneration, the implications for adult mammalian non-regenerative hearts are unclear. Zebrafish heart regeneration occurs by cardiomyocyte dedifferentiation and proliferation, both around the injury site and throughout the whole organ, as well as by cardiomyocyte migration^106–109^. This differs mechanistically from the adult mammalian response to cardiac injury, even if partial regeneration is induced. It is therefore difficult to extrapolate between species. On the other hand, comparative gene ontology suggested that species differences in ECM remodelling may be partly responsible for the different regenerative capacities^105^. Modification of injury-induced epicardial ECM remodelling might therefore present a method of converting the characteristics of the non-regenerative response towards those seen in regenerative hearts. Tissue engineering of pro-regenerative biomimetic matrices might be one approach to exploiting these novel findings^105^.

### The role of pericardial fluid

The pericardial sac enhances epicardial activation by constraining heart-derived signalling factors in close proximity to the epicardium^88^. Myocardially secreted FGF-1^110^ and FGF-2 levels^111^ in the pericardial fluid of MI patients were found to correlate with the severity of ischaemia and a possible role in mediating collateral growth, although a direct involvement of the epicardium in this process has not been explored.

A recent study in patients profiled the miRNA content of pericardial fluid and identified a number of previously implicated heart failure markers^112^; this was interpreted to be an active and selective paracrine mechanism, involving non-coding RNAs as well as growth factors, to mediate cross-talk between cardiac cell types, which likely includes epicardial cells. Administration of pericardial fluid into the pericardial cavity of non-infarcted mouse was sufficient to induce epicardial proliferation and re-expression of embryonic genes^88^.

Insulin-like growth factor 1 (IGF1), hepatocyte growth factor (HGF) and High mobility group box 1 protein (HMGB1), factors known to induce resident cardiac progenitors, were significantly elevated in pericardial fluid from MI patients^88^. Thus, pericardial fluid contains trophic factors which might be harnessed to invoke epicardial activation and repair. In a recent study, clusterin, was found to be secreted in exosomes of MI patients; addition of clusterin to the pericardial sac of mice post-MI enhanced epicardial EMT and arteriogenesis and led to improved cardiac function^113^. These studies demonstrate that intra-pericardial injection may be an effective delivery method for any future treatments to augment epicardial contribution to myocardial regeneration^114^.

### Translation into humans

The preclinical experiments described above have necessarily used non-human animal models, with the non-regenerative adult mouse heart implicitly assumed to be similar to adult human hearts. Nevertheless, experiments on human epicardial cells are required for translation of therapies into clinical practice. Several studies have used cultured primary epicardial cells, taken from right atrial appendages of human patients during right coronary artery bypass^115,116^; *in vitro* characterisation of the cells demonstrated basic similarities between mouse and human. Some species similarities were also noted in an *in situ* analysis of primary embryonic and foetal tissue samples^117^, in terms of marker expression and, broadly speaking, in its formation and apparent role during development. However, consistent differences were also reported between species both in fetal and adult epicardium; the embryonic human epicardium is not a simple squamous epithelium, as previously reported^118^. Instead, the external layer of flat mesothelial cells overlies a thin basal lamina with an underlying layer of connective tissue, the subepicardial space, containing elastic fibres as well as large vessels. In the adult human myocardium, the subepicardial space consists mainly of adipose tissue, which surrounds coronary vessels. By contrast, murine epicardium does not contain adipose tissue and comprises only a monolayer of mesothelial cells on a thin layer of connective tissue formed from elastic fibres^118^. These species differences suggest that experiments on human tissue may be important for translation from animal models to humans.

Furthermore, at least in the fetal human epicardium, atrial-ventricular differences in cellular behaviour were reported between epicardial cells. The ventricular, but not atrial, epicardium exhibited greater cell alignment and spindle-like morphology and *ex vivo* ventricular cells spontaneously differentiate and lose epicardial identity, whereas atrial-derived cells remained more ‘epithelial-like’^117^. The utility of cultured human atrial EPDCs as a model may therefore be limited not only by low availability but also by dissimilarity to the epicardial cells that may be stimulated *in vivo*. If atrial epicardial cells differ from ventricular epicardial cells, cells from atrial appendages may not necessarily represent the population of cells that partakes in the majority of reactivation after MI, albeit the extent of contribution *in vivo* from the individual chambers has not been addressed.

In an attempt to overcome these difficulties, epicardial-like cells have been generated from human pluripotent stem cells (hPSCs)^119^. One approach used stage-specific activation of bone morphogenetic protein (BMP) and WNT signalling pathways to generate cells with epicardial morphology expressing the epicardial markers *WT1*, *TBX18* and *RALDH2*. These epicardial-like cells could be induced to undergo EMT to produce populations displaying characteristics of fibroblast and vascular smooth muscle cell lineages^116^. An independent approach used a two-stage process to generate similar epicardial-like cells. The hPSCs were first converted to lateral plate mesoderm and then differentiated into epicardial-like cells by stimulation of BMP, WNT and retinoic acid signalling pathways^120^. These approaches can provide unlimited sources of cultured human epicardial-like cells which could be used to optimise potential treatments or could be combined with chemical genetic screening techniques to identify novel regenerative compounds and provide insight into poorly-understood signalling pathways^86,121^.

## Conclusion

High rates of cardiovascular disease mean that safe, effective and feasible cardiac regeneration could transform clinical practice. Reactivation of epicardial developmental processes is an attractive approach to coordinated regeneration of the heart and, if refined based on emerging concepts in epicardial biology, could provide novel strategies for regenerative epicardial reactivation.

## Funding sources

NS is supported by the British Heart Foundation Ian Fleming Senior Basic Science Research Fellowship.
